# Accuracy of molecular diagnostic methods for the detection of bovine brucellosis: A systematic review and meta-analysis

**DOI:** 10.14202/vetworld.2022.2151-2163

**Published:** 2022-09-08

**Authors:** Lerato Mabe, ThankGod E. Onyiche, Oriel Thekisoe, Essa Suleman

**Affiliations:** 1 NextGen Health Cluster, Council for Scientific and Industrial Research, P.O. Box 395, Pretoria, 0001, South Africa; 2 Unit for Environmental Sciences and Management, North-West University, Potchefstroom Campus, Private Bag X6001, Potchefstroom 2520, South Africa; 3 Department of Veterinary Parasitology and Entomology, University of Maiduguri, P. M. B. 1069, Maiduguri 600230, Nigeria

**Keywords:** bovine brucellosis, *Brucella abortus*, meta-analysis, molecular diagnosis, systematic review

## Abstract

**Background and Aim:**

Bovine brucellosis is a disease of global socio-economic importance caused by *Brucella abortus*. Diagnosis is mainly based on bacterial culture and serology. However, these methods often lack sensitivity and specificity. A range of molecular diagnostic methods has been developed to address these challenges. Therefore, this study aims to investigate the diagnostic accuracy of molecular tools, in comparison to gold standard bacterial isolation and serological assays for the diagnosis of bovine brucellosis.

**Materials and Methods:**

The systematic review and meta-analysis were conducted based on analyses of peer-reviewed journal articles published between January 1, 1990, and June 6, 2020, in the PubMed, Science Direct, Scopus, and Springer Link databases. Data were extracted from studies reporting the use of molecular diagnostic methods for the detection of *B. abortus* infections in animals according to Preferred Reporting Items for Systematic Reviews and Meta-analyses (PRISMA) guidelines. The quality of included journal articles was assessed using the quality assessment of diagnostic-accuracy studies assessment tool and meta-analysis was carried out using Review Manager.

**Results:**

From a total of 177 studies, only 26 articles met the inclusion criteria based on PRISMA guidelines. Data from 35 complete studies were included in the meta-analysis and used to construct 2 × 2 contingency tables. Improved diagnostic performance was observed when tissue (sensitivity 92.7% [95% confidence interval (CI) 82.0–98.0%]) and serum samples (sensitivity 91.3% [95% CI 86.0–95.0%]) were used, while the BruAb2_0168 locus was the gene of preference for optimal assay performance (sensitivity 92.3% [95% CI 87.0–96.0%] and specificity 99.3% [95% CI 98.0–100.0%]). Loop-mediated isothermal amplification (LAMP) had a higher diagnostic accuracy than polymerase chain reaction (PCR) and real-time quantitative PCR with sensitivity of 92.0% (95% CI 78.0–98.0%) and specificity of 100.0% (95% CI 97.0–100.0%).

**Conclusion:**

The findings of this study assign superior diagnostic performance in the detection of *B. abortus* to LAMP. However, due to limitations associated with decreased specificity and a limited number of published articles on LAMP, the alternative use of PCR-based assays including those reported in literature is recommended while the use of LAMP for the detection of bovine brucellosis gains traction and should be evaluated more comprehensively in future.

## Introduction

Brucellosis is a re-emerging infectious and communicable disease of livestock known for its significant impact on global human health and economy [1]. It is caused by Gram-negative, facultative intracellular bacteria of the genus *Brucella* [2]. Specifically, *Brucella abortus* and *Brucella melitensis* are the key causal agents of brucellosis in bovids with a greater association with cattle attributed to *B. abortus* [3]. The disease is easily spread through the movement of infected cattle and direct contact with infected livestock constitutes a risk factor for human transmission [4, 5]. However, consumption of contaminated meat and unpasteurized dairy products is the primary route of infection in humans [6]. The clinical presentation of brucellosis is not distinctly indicative of infection with *Brucella* except for third trimester abortion in pregnant animals [7]. A definitive diagnosis is almost entirely reliant on the combined use of bacteriological and serological methods [8]. An assembled diagnostic approach is necessary due to the prevailing limitations of these techniques. While bacterial isolation and identification result in a definitive diagnosis, this is rather impractical for testing large herds [9]. Serological methods are comparatively faster, easier, and safer, and therefore have been favorably used for mass screening of infectious bacteria [10]. However, their cross-reactivity with other microorganisms that share the same smooth lipopolysaccharides, often lead to a false positive diagnosis, while the delayed production of antibodies in early infection can lead to misdiagnosis [11].

To address the challenges associated with bacterial and serological identification, various molecular diagnostic assays have been developed for the detection of *B. abortus* with polymerase chain reaction (PCR)-based assays being the more dominant technology [12–15]. These techniques are believed to have significant advantages over traditional methods and have become desired tools for application in disease diagnostics. However, for these diagnostic tools to support accurate and timely diagnosis of bovine brucellosis, and possibly even replace the use of serological tests at the point-of-care, they need to display high diagnostic accuracy (sensitivity and specificity). With increasing applicability for the detection of infectious pathogens, careful evaluation of these diagnostic assays against gold standard techniques, must precede their implementation on larger scales.

To the best of our knowledge, there is currently no systematic review that explores the diagnostic accuracy of molecular methods for the detection of *B. abortus*. Therefore, this study aims to investigate the diagnostic accuracy of molecular tools, in comparison to gold standard bacterial isolation and serological assays for the diagnosis of bovine brucellosis.

## Materials and Methods

### Ethical approval

This is a systematic review; therefore ethical approval was not required. Reporting was based on the Preferred Reporting Items for Systematic Reviews and Meta-Analyses (PRISMA) 2020 statement: An updated guideline for reporting systematic reviews [16].

### Study period and location

The systematic review took place between March 2020 and August 2020. It was carried out in Pretoria, South Africa, at the Council for Scientific and Industrial Research, Veterinary Molecular Diagnostics and Vaccines group.

### Study scope and definition of the gold standard

This study was undertaken to assess the accuracy of molecular diagnostic methods for detection of *B. abortus* with gold standard methods of detection defined as positive bacterial isolation and identification of the causal agent as *B. abortus* and/or positive reaction to one or more serological tests.

### Search strategy and selection

Online searches for relevant published material were carried out on the PubMed, Science Direct, Scopus, and Springer Link databases. The searches included all articles published from January 1, 1990, to June 6, 2020. A combination of the following search terms was used to search the databases: “*B. abortus*,” “Bovine Brucellosis,” “Molecular Diagnosis,” “Molecular Detection,” “PCR,” “real-time PCR,” “multiplex PCR,” “recombinase polymerase amplification,” and “loop-mediated isothermal amplification (LAMP).” These key terms were used either individually or in combination with “AND” and/or “OR” operators. Articles were screened for relevance and downloaded after assessment of their titles and abstracts. A thorough search of the reference lists of each of the downloaded articles was conducted to find additional publications of relevance.

### Study inclusion/exclusion criteria

Title screening was the first criterion for the inclusion of articles in this study. This was followed by the screening of abstracts and a comprehensive assessment of the contents of each research article by thorough scrutiny of the full text. On full-text review, the following inclusion criteria were applied: (i) Study employs the use of molecular diagnostic techniques for detection of bovine brucellosis, (ii) study detailing specific detection of *B. abortus* or the differentiation of *B. abortus* from other species of *Brucella*, (iii) study involving the comparison of molecular techniques with either or both of the gold standard tests as per the definition of the gold standard provided herein, (vi) full-text article available in English, (vi) assays validated on clinical samples collected from large ruminants, and (vii) articles in which two-by-two contingency tables could be completed. Any other study that did not meet the inclusion criteria specified above was excluded from our study. Study inclusion was independently conducted by two of the authors (LM and TEO); where decisive action could not be taken, a third independent investigator was approached for a final decision.

### Data extraction

The following data were extracted from each of the studies that were included in this systematic review and meta-analysis investigating the diagnostic accuracy of molecular assays for the detection of *B. abortus*: (i) Citations, (ii) study area, (iii) index test assessed, (iv) targeted gene region (v) type of sample used, and (vi) type of gold standard test used. The extracted data were entered into an excel spreadsheet and used to generate 2 × 2 contingency tables. All studies from which a 2 × 2 contingency table could not be completed were excluded from the meta-analysis.

### Quality assessment of included studies

Quality assessment of the included studies was performed using the quality assessment of diagnostic-accuracy studies tool (QUADAS-2) [17].

### Data analysis and synthesis

Sensitivity and specificity estimates together with their 95% confidence intervals (CI) were computed using MedCalc^®^ statistical software version 13.0.2 for Windows (MedCalc Software Bvba, Ostend, Belgium; http://www.medcalc.org) and plotted on forest plots and on Summary Receiver Operator Characteristic curves (SROC) using Review Manager (Cochrane Collaboration, London, UK). To compare the diagnostic accuracy between the two assays, we used four likelihood ratios namely, PLR: Positive likelihood ratio, NLR: Negative likelihood ratio, PPV: Positive predictive value; and NPV: Negative predictive value. This analysis was carried out using the MedCalc^®^ statistical software version 13.0.2 for Windows (MedCalc Software Bvba; http://www.medcalc.org).

Pooled estimate values for sensitivity and specificity were also computed in MedCalc^®^. Subgroup analyses were performed to determine diagnostic accuracy based on the following possible factors: (i) The index test used, (ii) the type of clinical sample tested, and (iii) the choice of target gene. A Chi-square test was performed in excel to determine the association between studies. Studies were regarded significantly different when p-values were <0.05 (p < 0.05).

## Results

### Literature search and included studies

**Figure-1:**
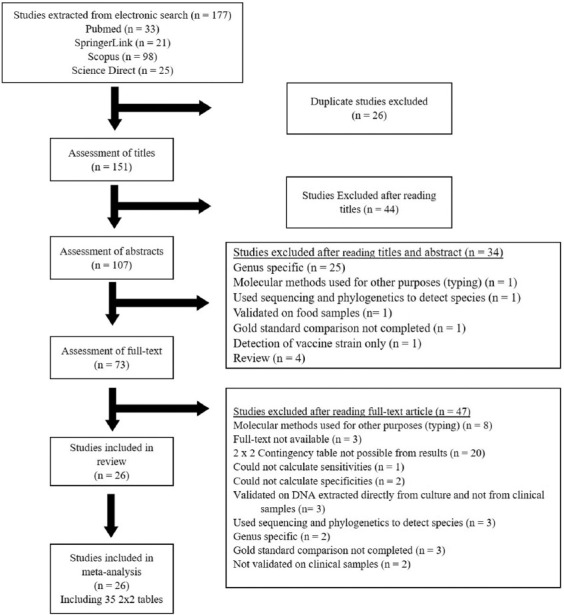
Flowchart of included studies adapted from PRISMA by Page *et al*. [16].

The electronic databases search identified 177 articles (Figure-1) [16] based on primary search criteria. Of these 177 articles, 26 duplicate articles were excluded, leaving 151 articles. A further 44 articles were excluded after reading the title resulting in 107 studies which were suitable for assessment of the abstracts. Of these, 34 studies were excluded, as detailed in Figure-1. The 73 remaining articles were assessed for eligibility through an in-depth assessment of the full text. From these, 47 articles were excluded as follows: (1) Full-text not available (or not available in English) (n = 3), (2) molecular methods used for purposes other than diagnostics (e.g., genotyping) (n = 8), (3) not possible to construct a 2 × 2 contingency table from results (n = 20), (4) could not calculate sensitivities and/or specificities (n = 3), (5) assays not validated on clinical samples (or samples collected from either large or small ruminants) (n = 5), (6) assays which did facilitate species identification (genus-specific assay or use of sequencing and phylogenetics for species identification) (n = 2), (7) used sequencing or phylogenetics for species identification (n = 3), and (8) no baseline test used for comparison (gold standard) (n = 3). Twenty-six articles were consistent with the inclusion criteria and therefore selected for inclusion in the systematic review and meta-analysis.

### Characteristics of included studies

The index tests assessed in the included studies were PCR (n = 23), real-time PCR (n = 10), and LAMP (n = 2). The studies were validated on extracted DNA from whole blood (n = 1), serum samples (n = 3), milk (n = 8), tissue samples (n = 4), vaginal exudates or semen (n = 3) as well as mixed samples (n = 7). The assays used in these studies targeted specific regions of the *B. abortus* genome including the BCSP31 gene (n = 3), BruAb2_0168 locus (n = 5), IS711 genetic element (23), and other genes (n = 4) (Table-1) [1, 3, 5, 7, 9, 12–15, 18–35].

**Table-1:** Characteristics of included studies.

Study enrolled	Country	Index test	Target gene	Clinical sample	Study design	Reference standard
Alamian *et al*. [12]	Iran	PCR	Polymorphic repeat sequence region in chromosome 1 of *B. abortus*	Animal blood and Lymph nodes	Cross-sectional	Isolation
Akoko *et al*. [20]	Kenya	qPCR	IS711 element downstream of alkB gene	Serum	Cross-sectional	Serology
Ali *et al*. [23]	Pakistan	qPCR	IS711 element downstream of alkB gene	Blood	Cross-sectional	Serology
Arellano- Reynoso *et al*. [15]	Mexico	PCR	IS711 element downstream of wboA gene	Milk	Cross-sectional	Isolation
Arellano- Reynoso *et al*. [15]	Mexico	PCR	IS711 element downstream of wboA gene	Vaginal exudates	Cross-sectional	Isolation
Barkallah *et al*. [26]	Tunisia	qPCR	IS711 element downstream of alkB gene	Blood	Cross-sectional	Serology
Barkallah *et al*. [26]	Tunisia	qPCR	IS711 element downstream of alkB gene	Vaginal swab	Cross-sectional	Serology
Barkallah *et al*. [26]	Tunisia	qPCR	IS711 element downstream of alkB gene	Milk	Cross-sectional	Serology
de Oliveira *et al*. [30]	Pantanal	PCR	BruAb2_0168 locus	Blood	Cross-sectional	Serology
Chisi *et al*. [9]	South Africa	PCR	IS711 element downstream of alkB gene	Milk, Abomasal fluid from aborted foetuses, Uterine discharge, Hygroma fluid, Lymph nodes	Cross-sectional	Isolation
Dehkordi *et al*. [25]	Iran	qPCR	BruAb2_0168 locus	Semen	Cross-sectional	Isolation
Doust *et al*. [1]	Iran	PCR	IS711 element downstream of alkB gene	Tissue	Cross-sectional	Isolation
Gwida *et al*. [31]	Egypt	qPCR	IS711 element downstream of alkB gene	Serum	Cross-sectional	Serology
Hamdy and Amin [32]	Egypt	PCR	IS711 element downstream of alkB gene	Milk	Cross-sectional	Isolation
Kang *et al*. [5]	South Korea	LAMP	BruAb2_0168 locus	Tissue and buffy coats	Cross-sectional	Isolation
Kang *et al*. [5]	South Korea	qPCR	BruAb2_0168 locus	Tissue and buffy coats	Cross-sectional	Isolation
Karthik *et al*. [7]	India	LAMP	BruAb2_0168 locus	Whole blood and aborted fetal stomach contents	Cross-sectional	Serology
Karthik *et al*. [29]	India	PCR	IS711 element downstream of alkB gene	Serum and blood	Cross-sectional	Serology
Kaynak-Onurdag *et al*. [33]	Turkey	qPCR	Outer membrane protein	Milk	Cross-sectional	Isolation
Leal-Klevezas *et al*. [21]	Mexico	PCR	Unspecified	Blood	Cross-sectional	Isolation
Leal-Klevezas *et al*. [21]	Mexico	PCR	Unspecified	Blood	Cross-sectional	Serology
Nardi Júnior *et al*. [24]	Brazil	PCR	IS711 element downstream of wboA gene	Serum and Semen	Cross-sectional	Isolation
Narnaware *et al*. [18]	India	PCR	IS711 element downstream of alkB gene	Blood	Cross-sectional	Serology
Narnaware *et al*. [18]	India	PCR	IS711 element downstream of alkB gene	Tissue	Cross-sectional	Serology
Naz *et al*. [27]	Pakistan	PCR	IS711 element downstream of wboA gene	Blood	Cross-sectional	Serology
Naz *et al*. [27]	Pakistan	PCR	IS711 element downstream of wboA gene	Blood	Cross-sectional	Serology
Neha *et al.* [19]	India	PCR	IS711 element downstream of alkB gene	Blood	Cross-sectional	Serology
Ning *et al*. [14]	China	PCR	IS711 element downstream of alkB gene	Milk	Cross-sectional	Serology
O’ Leary *et al*. [3]	Ireland	PCR	IS711 element downstream of alkB gene	Lymph node tissue	Cross-sectional	Isolation
O’ Leary *et al*. [3]	Ireland	PCR	IS711 element downstream of alkB gene	Milk	Cross-sectional	Isolation
Rani *et al*. [34]	India	PCR	BCSP31 gene	Blood	Cross-sectional	Serology
Sreevatsan *et al*. [13]	India and USA	PCR	BCSP31 gene	Liver	Cross-sectional	Isolation
Sreevatsan *et al*. [13]	India and USA	PCR	BCSP31 gene	Milk	Cross-sectional	Serology
Terzi *et al*. [22]	Turkey	PCR	IS711 element downstream of alkB gene	Milk	Cross-sectional	Serology
Wareth *et al*. [28]	Egypt	qPCR	IS711 element downstream of alkB gene	Serum	Cross-sectional	Serology

USA=United States of America, LAMP=Loop-mediated isothermal amplification, PCR=Polymerase chain reaction, qPCR=Quantitative real-time PCR

### Methodological quality of included studies

The methodological qualities of included studies were assessed using the QUADAS-2 assessment tool and the results are presented in Table-2 [3, 5, 7, 9, 12–15, 18–35]. The major risk of bias was identified in the patient selection category where six studies were identified to be at high risk of bias [3, 7, 18, 19, 35]. In these studies, patient enrollment was based on prior knowledge of the disease status of selected patients due to prior testing or suspected cases selected based on clinical presentation suggestive of brucellosis. In this category, only four studies were identified to be at low risk of bias [20–22, 25]. For the remaining 18 studies, patient enrollment was not defined sufficiently to determine if the selection was random or consecutive. In the index test domain, two studies were considered to be at high risk of bias [20, 23]. In these studies, the index test was conducted with knowledge of the results of the gold standard test, leading to the exclusion of gold standard negative samples from analysis with the index test which may have introduced bias. In the reference standard domain, it was unclear if the results of the gold standard were interpreted without knowledge of the results of the index test. However, in most of these studies all the samples were subjected to gold standard testing and the results were interpreted independently; therefore, it was inferred that these presented a low risk of bias. Concern for applicability in the patient selection category was unclear; however, there was little concern that the index test, its conduct and interpretation or the target condition as defined by the gold standard test do not match the review question.

**Table-2:** A summary of methodological qualities of included studies determined for each domain of QUADAS-2.

Study enrolled	Risk of Bias				Applicability Concerns		
	Patient selection	Index test	Reference standard	Flow and timing	Patient selection	Index test	Reference standard
Alamian *et al*. [12]	?	+	+	?	?	+	+
Akoko *et al*. [20]	+	-	+	?	+	+	+
Ali *et al*. [23]	?	-	+	?	?	+	+
Arellano-Reynoso *et al*. [15]	?	?	+	?	?	+	+
Barkallah *et al*. [26]	?	+	+	?	?	+	+
de Oliveira *et al*. [30]	?	?	+	?	?	+	+
Chisi *et al*. [9]	?	+	+	?	?	+	+
Dehkordi *et al*. [25]	+	+	?	?	+	+	+
Doust *et al*. [35]	-	+	+	?	-	+	+
Gwida *et al*. [31]	?	+	+	?	?	+	+
Hamdy and Amin [32]	?	+	+	?	?	+	+
Kang *et al*. [5] (A)	?	+	?	?	?	+	+
Kang *et al*. [5] (B)	?	?	?	?	?	+	+
Karthik *et al*. [7] a	-	+	+	?	-	+	+
Karthik *et al*. [29] b	?	+	+	?	?	+	+
Kaynak-Onurdag *et al*. [33]	?	+	+	?	?	+	+
Leal-Klevezas *et al*. [21]	+	+	+	?	+	+	+
Nardi Júnior *et al*. [24]	?	+	+	?	?	+	+
Narnaware *et al*. [18]	-	+	+	?	-	+	+
Naz *et al*. [27]	?	+	+	?	?	+	+
Neha *et al*. [19]	-	?	+	?	-	+	+
Ning *et al*. [14]	?	+	?	?	?	+	+
O’ Leary *et al*. [3]	-	?	+	?	-	+	+
Rani *et al*. [34]	?	?	?	?	?	+	+
Sreevatsan *et al*. [13]	?	+	+	?	?	+	+
Terzi *et al*. [22]	+	+	+	?	+	+	+
Wareth *et al*. [28]	-	+	+	?	-	+	+

QUADAS-2=Quality assessment of diagnostic-accuracy studies tool, +=Low, -=High, ?=Unclear. (A) and (B) refer to different sets of data obtained from two different index tests within the same publication, whereas small letters a and b indicate different publications

### Diagnostic accuracy of molecular assays

**Figure-2:**
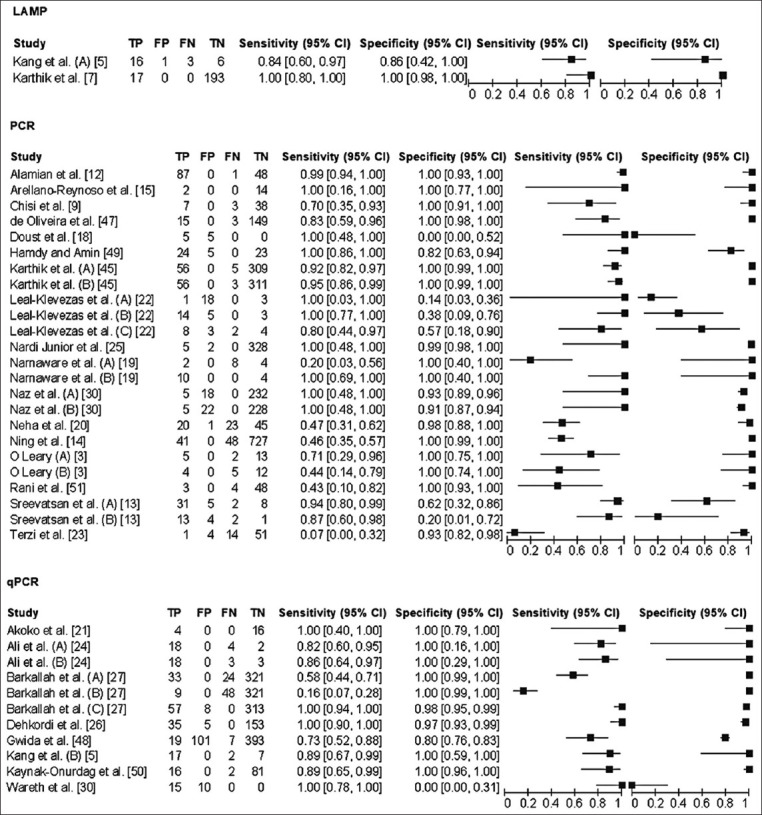
Forest plot with sensitivity and specificity estimates of the included studies with respect to different index tests (Loop-mediated isothermal amplification, polymerase chain reaction (PCR) and quantitative PCR. Capital (A)-(C) indicate different sets of data from the same publication. These data sets may differ in the clinical sample, index test or gold standard test used.

**Figure-3:**
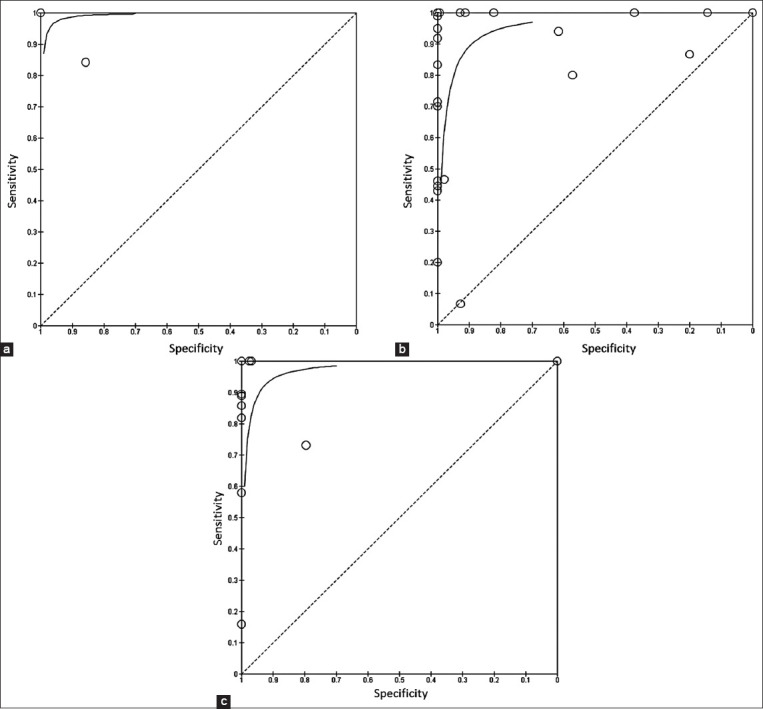
(a) Summary Receiver Operator Characteristic curve showing the sensitivity and specificity estimates for all the included studies using loop-mediated isothermal amplification, (b) polymerase chain reaction (PCR), and (c) quantitative PCR (qPCR).

The 26 eligible citations that were included in this study represent a total of 35 separate studies with their complete 2 × 2 contingency tables. The sensitivities reported by these studies range from 7.0% to 100.0% while the specificities reported were in the range of 0.0–100.0%, these together with the diagnostic accuracy measures of the respective studies are reported (Table-3) [3, 5, 7, 9, 12–15, 18–35]. Graphical representation of the sensitivity and specificity estimates for each study are represented on forest plots (Figure-2) [1–5, 7, 9, 12–33, 35, 36] and SROC curves (Figure-3). Of the 35 studies that were included in the meta-analysis, only two analyzed the accuracy of LAMP for detection of bovine brucellosis. Pooled sensitivity and specificity estimates for LAMP were 91.7% (95% CI: 78.0–98.0%) and 99.5% (95% CI: 97.0–100.0%), respectively. Among the two studies that were reported, Karthik *et al*. [7] reported an assay with higher sensitivity (100% [95% CI: 80.0–100.0%]) and specificity (100% [95% CI: 98.0–100.0%]). Twenty-four studies evaluated the use of PCR, which yielded pooled sensitivity and specificity estimates of 77.1% [95% CI 73.0–81.0%] and 96.6% [95% CI 96.0–97.0%], respectively. Of these, the studies reported Arellano-Reynoso *et al*. [15] and Narnaware *et al*. [18] both reported 100% sensitivity and specificity estimates while Alamian *et al*. [12] and Nardi Jr. *et al*. [24] both reported 99.0% sensitivity and 100% specificity (Table-3 for 95% CIs). Eleven studies evaluated the accuracy of quantitative PCR (qPCR) with pooled sensitivity and specificity values of 72.8% (95% CI 68.0–78.0%) and 92.8% (95% CI 92.0–94.0%), respectively (Table-4). Of these Akoko *et al*. [20] (sensitivity 100.0% [95% CI: 44.0–71.0%] and specificity 100% [95 CI: 99.0–100.0%]), Dehkordi *et al*. [25] (sensitivity 100.0% [95% CI: 44.0–71.0%] and specificity 100% [95 CI: 99.0–100.0%]), and Barkallah *et al*. [26] (sensitivity 100.0% [95% CI: 44.0–71.0%] and specificity 100% [95 CI: 99.0–100.0%]), are worth mentioning. Further analysis of subgroups was performed to determine the diagnostic accuracy of molecular assays when they are applied to different sample types and gene targets. The pooled estimates for the sensitivity and specificity of these subgroups are also presented in Table-4. Based on Chi-square analysis significant differences were observed in the diagnostic accuracy (sensitivity and specificity) of the three index tests (LAMP, PCR, and qPCR), and in the diagnostic accuracy of the assays based on the type of sample used and the gene targets (Table-4). According to the data presented herein, we found that a large number of true *B. abortus* infections could be detected using tissue samples compared to serum samples. The diagnostic specificity for these sample choices was, however, lower (71.4% [95% CI 54.0–85.0%] and 80.1% [95% CI 77.0–83.0%], respectively), compared to those of other samples.

**Table-3:** Diagnostic accuracy measures for all included studies.

Indextest	Study enrolled	TP	FP	FN	TN	PPV	NPV	LR+	LR-	Sensitivity	Specificity
LAMP	Kang *et al*. [5]	16	1	3	6	0.94	0.67	5.89	0.18	0.84 (0.60–0.97)	0.86 (0.42–1.00)
	Karthik *et al*. [7]	17	0	0	193	1.00	1.00	NE	0.00	1.00 (0.80–1.00)	1.00 (0.98–1.00)
PCR	Alamian *et al*. [12]	87	0	1	48	1.00	0.98	NE	0.01	0.99 (0.94–1.00)	1.00 (0.93–1.00)
	Arellano-Reynoso *et al*. [15]	2	0	0	14	1.00	1.00	NE	0.00	1.00 (0.16–1.00)	1.00 (0.77–1.00)
	de Oliveira *et al*. [30]	15	0	3	149	1.00	0.98	NE	0.17	0.83 (0.59–0.96)	1.00 (0.98–1.00)
	Chisi *et al*. [9]	7	0	3	38	1.00	0.93	NE	0.30	0.70 (0.35–0.93)	1.00 (0.91–1.00)
	Doust *et al*. [35]	5	5	0	0	0.50	NE	1.00	NE	1.00 (0.48–1.00)	0.00 (0.00–0.52)
	Hamdy and Amin [32]	24	5	0	23	0.83	1.00	5.60	0.00	1.00 (0.86–1.00)	0.82 (0.63–0.94)
	Karthik *et al*. [29] (A) b	56	0	5	309	1.00	0.98	NE	0.08	0.92 (0.82–0.97)	1.00 (0.99–1.00)
	Karthik *et al*. [29] (B) b	56	0	3	311	1.00	0.99	NE	0.05	0.95 (0.86–0.99)	1.00 (0.99.1.00)
	Leal-Klevezas *et al*. [21] (A)	1	18	0	3	0.05	1.00	1.17	0.00	1.00 (0.03–1.00)	0.14 (0.04–0.36)
	Leal-Klevezas *et al*. [21] (B)	14	5	0	3	0.74	1.00	1.60	0.00	1.00 (0.77–1.00)	0.38 (0.09–0.76)
	Leal-Klevezas *et al*. [21] (C)	8	3	2	4	0.73	0.67	1.87	0.35	0.80 (0.44–0.97)	0.57 (0.18–0.90)
	Nardi Júnior *et al*. [24]	5	2	0	328	0.71	1.00	165.0	0.00	1.00 (0.48–1.00)	0.99 (0.98–1.00)
	Narnaware *et al*. [18] (A)	2	0	8	4	1.00	0.33	NE	0.80	0.20 (0.03–0.56)	1.00 (0.40–1.00)
	Narnaware *et al*. [18] (B)	10	0	0	4	1.00	1.00	NE	0.00	1.00 (0.69–1.00)	1.00 (0.40–1.00)
	Naz *et al*. [27] (A)	5	18	0	232	0.22	1.00	13.89	0.00	1.00 (0.48–1.00)	0.93 (0.89–0.96)
	Naz *et al*. [27] (B)	5	22	0	228	0.19	1.00	11.36	0.00	1.00 (0.48–1.00)	0.91 (0.87–0.94)
	Neha *et al*. [19]	20	1	23	45	0.95	0.66	21.39	0.55	0.47 (0.31–0.62)	0.98 (0.88–1.00)
	Ning *et al*. [14]	41	0	48	727	1.00	0.94	NE	0.54	0.46 (0.35–0.57)	1.00 (0.99–1.00)
	O’ Leary *et al*. [3] (A)	5	0	2	13	1.00	0.87	NE	0.29	0.71 (0.29–0.96)	1.00 (0.75–1.00)
	O’ Leary *et al*. [3] (B)	4	0	5	12	1.00	0.71	NE	0.56	0.44 (0.14–0.79)	1.00 (0.74–1.00)
	Rani *et al*. [34]	3	0	4	48	1.00	0.92	NE	0.57	0.43 ( 0.10–0.82)	1.00 (0.93–1.00)
	Sreevatsan *et al*. [13] (A)	31	5	2	8	0.76	0.33	1.08	0.67	0.94 (0.80–0.99)	0.62 (0.32–0.86)
	Sreevatsan *et al*. [13] (B)	13	4	2	1	0.86	0.80	2.44	0.09	0.87 (0.60–0.98)	0.20 (0.01–0.72)
	Terzi *et al*. [22]	1	4	14	51	0.20	0.78	0.92	1.01	0.07 (0.02–0.32)	0.93 (0.82–0.98)
qPCR	Akoko *et al*. [20]	4	0	0	16	1.00	1.00	NE	0.00	1.00 (0.44–0.71)	1.00 (0.99–1.00)
	Ali *et al*. [23] (A)	18	0	4	2	1.00	0.33	NE	0.18	0.82 (0.07–0.82)	1.00 (0.99–1.00)
	Ali *et al*. [23] (B)	18	0	3	3	1.00	0.50	NE	0.14	0.86 (0.40–1.00)	1.00 (0.79–1.00)
	Barkallah *et al*. [26] (A)	33	0	24	321	1.00	0.93	NE	0.42	0.58 (0.60–0.95)	1.00 (0.16–1.00)
	Barkallah *et al*. [26] (B)	9	0	48	321	1.00	0.87	NE	0.84	0.16 (0.64–0.97)	1.00 (0.29–1.00)
	Barkallah *et al*. [26] (C)	57	8	0	313	0.88	1.00	40.13	0.00	1.00 (0.94–1.00)	0.98 (0.95–0.99)
	Dehkordi *et al*. [25]	35	5	0	153	0.88	1.00	31.60	0.00	1.00 (0.90–1.00)	0.97 (0.93–0.99)
	Gwida *et al*. [31]	19	101	7	393	0.16	0.98	3.57	0.39	0.73 (0.52–0.88)	0.80 (0.76–0.83)
	Kang *et al*. [5]	17	0	2	7	1.00	0.78	NE	0.10	0.89 (0.67–0.97)	1.00 (0.59–1.00)
	Kaynak-Onurdag *et al*. [33]	16	0	2	81	1.00	0.98	NE	0.11	0.89 (0.65–0.98)	1.00 (0.96–1.00)
	Wareth *et al*. [28]	15	10	0	0	0.60	NE	1.00	NE	1.00 (0.78–1.00)	0.00 (0.00–0.31)

LAMP=Loop-mediated isothermal amplification, qPCR=Quantitative polymerase chain reaction, TP=True positive; FP=False positive; FN=False negative; TN=True negative, PPV=Positive predictive value, NPV=Negative predictive value, LR+=Likelihood ratio positive, LR-=Likelihood ratio negative, NE=Not estimated. (A), (B), and (C) refer to different sets of data from the same publication, whereas small letters a and b indicate different publications.

**Table-4:** Summary estimates for the different index tests, sample types, and target genes.

Factor	Number of studies	Pooled sensitivity (95% CI)	Pooled specificity (95% CI)
Index test			
LAMP	2	0.92 (0.78–0.98)	1.00 (0.97–1.00)
PCR	22	0.77 (0.73–0.81)	0.97 (0.96–0.97)
qPCR	10	0.73 (0.68–0.78)	0.93 (0.92–0.94)
p-value		0.0016	0.0218
Sample type			
Blood	10	0.61 (0.53–0.69)	0.94 (0.93–0.95)
Milk	8	0.69 (0.62–0.75)	0.98 (0.97–0.99)
Serum	3	0.91 (0.86–0.95)	0.80 (0.77–0.83)
Tissue	4	0.93 (0.82–0.98)	0.71 (0.54–0.85)
Vaginal exudates or semen	3	0.49 (0.38–0.59)	0.99 (0.97–1.00)
p-value		<0.0001	<0.0001
Target gene			
BCSP31 gene	3	0.85 (0.73–0.94)	0.86 (0.76–0.94)
BruAb2_0168 locus	5	0.92 (0.87–0.96)	0.99 (0.98–1.00)
IS711 genetic element	23	0.70 (0.65–0.73)	0.95 (0.94–0.96)
p-value		0.0002	0.0008

CI=Confidence interval, LAMP=Loop-mediated isothermal amplification, q-PCR=Quantitative polymerase chain reaction

## Discussion

The culture and isolation of bacterial disease agents remain the gold standard tool for the diagnosis of bovine brucellosis. However, due to a myriad of drawbacks, including decreased diagnostic efficiency associated with requirements for extended culture periods, stringent laboratory conditions, highly skilled personnel, serology remains an integral part of the brucellosis testing regimen [9], despite its limited specificity due to shared common antigenic lipopolysaccharides between species of *Brucella* that result in cross-reactivity during testing [11]. Molecular assays (PCR, qPCR, and LAMP) have a higher degree of sensitivity and specificity and facilitate differentiation between members of the genus *Brucella* [7, 37]. Therefore, this review sought to assess the diagnostic accuracy of molecular assays in detecting *B. abortus* induced infections in bovines (cattle), to inform decision-making and encourage the adoption of these techniques as alternatives to conventional methods of diagnosis.

To assess the diagnostic accuracy, we first considered the methodological qualities of the studies that were selected for inclusion in this meta-analysis. The analysis found that the overall risk of bias was unclear while the concern for applicability was low. In some studies, however [7, 15, 18, 20, 21, 23, 27, 38], there were subjects that did not receive the index test, which poses a risk for the potential exclusion of data and risk of reporting bias. In addition, in the study reported by Arellano-Reynoso *et al*. [15], many subjects did not receive both the gold standard and the index test, and those that were subjected to index testing were not subjected to the same gold standard which could have introduced bias.

The pooled sensitivity and specificity estimates showed that both the diagnostic sensitivity and specificity across index tests were high for molecular diagnostic assays, suggesting superiority over traditional bacterial culture and serological assays. than agnostic sensitivity (72.8–91.7%) was lower compared to specificity (92.8–99.5%). These higher diagnostic specificities can possibly be attributed to the improved diagnostic specificity of molecular tests compared to traditional bacterial culture and serological assays which were the reference standards in this study. Limitations in the accuracy of a gold standard test in detecting a specific target condition can result in the lower diagnostic sensitivity of the index test if the gold standard test is used as a benchmarking tool to judge the performance of the index test. For instance, in 2015 Wareth *et al*. [28] reported that the detection of *B. abortus* in small ruminants using real-time PCR was far more superior (100% detection rate) compared with serological methods (Rose Bengal Test, Complement Fixation Test and Enzyme-linked immunosorbent assays) which were able to detect *B. abortus* infections in only 40% of the samples collected from abortion events while bacteriological methods failed to isolate *Brucella* spp. from any of the samples that were brought in for testing. Taking this into consideration, we can assume that some cases that were identified as negatives in some of the studies included in this review (particularly serum and tissue-based studies) are possibly false negatives and in fact may be true *B. abortus* cases that could not be picked up by gold standard tests owing to the low diagnostic sensitivity of these assays.

In a similar systematic review on molecular tools for diagnosing visceral leishmaniasis, de Ruiter *et al*. [39], also cite inferior gold standard accuracy as a possible reason for the lack of congruence in their diagnostic accuracy data. Therefore, the lower diagnostic specificity of molecular tests in this review can be explained by the limited sensitivity of both the culture method and serological tests. With classical microbiological methods, the probability of successfully isolating *B. abortus* in any given sample is significantly reduced in the early stages of infection where bacterial numbers are low and in instances where a sample may be heavily contaminated [28]. Similarly, with serology, antibody titers to *B. abortus* may take up to 2 weeks to rise to detectable levels after infection [21, 40]. Therefore early-stage infection testing may fail to pick up positive infections due to the absence of detectable antibody titers in the serum of an infected host. The same effect can be induced in chronic infections, with antibody titers falling below detectable levels [41]. Furthermore, some infections may remain dormant in the host and therefore, not seroconvert, further complicating the diagnostic process [42]. These factors may explain the low specificity values observed in our study, suggesting inferiority of gold standard tests in picking up infections that were otherwise detected by molecular tools, and in the respective individual studies, were regarded as negative cases but are possibly missed true positive cases of *B. abortus* infection.

The results presented herein suggest that LAMP, having higher pooled diagnostic accuracy estimates than PCR and qPCR, has a superior diagnostic performance to the two. Among the LAMP assays included in the meta-analysis the assay reported in Karthik *et al*. [7], was found to be of higher diagnostic accuracy (sensitivity 100.0% [95% CI 80.0–100.0%] and specificity 100.0% [95% CI 98.0–100.0%]) thus, can be recommended for detection of bovine brucellosis where LAMP is the desired assay. When the PCR subgroup was evaluated based on sensitivity and specificity estimates, assays reported by Alamian *et al*. [12], Arellano-Reynoso *et al*. [15], Narnaware *et al*. [18], and Nardi Jr. *et al*. [24], had higher diagnostic accuracy than the rest. Of the four studies, Arellano-Reynoso *et al*. [15] and Narnaware *et al*. [18] reported 100% sensitivity and specificity but the confidence levels in these assays were low compared to those reported in Alamian *et al*. [12] and Nardi Jr. *et al*. [24] (Table-3). Consequently, the PCR assay reported in Alamian *et al*. [12] is recommended as an alternative molecular diagnostic assay where PCR is the assay of choice due to the higher confidence level reported than that reported in Nardi Jr. *et al*. [24], who reported similar sensitivity and specificity values. When the qPCR subgroup was considered, assays reported in Akoko *et al*. [20], Dehkordi *et al*. [25], and Barkallah *et al*. [26], stood out from the rest in terms of diagnostic accuracy. Although Akoko *et al*. [20] reported 100% sensitivity and specificity, due to the low confidence levels reported for the sensitivity of this assay, we recommend the assays reported in Dehkordi *et al*. [25] and Barkallah *et al*. [26] for accurate detection of bovine brucellosis where qPCR is the desired alternative molecular diagnostic test.

Research ascribes better diagnostic performance to isothermal amplification techniques than their PCR-based counterparts [7, 43]. The superior performance of LAMP is particularly attributed to the use of four to six primers that bind to six to eight unique regions of the target gene [44]. However, while earlier studies have demonstrated the superiority of LAMP over PCR [5, 7, 43, 45–48], some [5, 48] have contrarily indicated a lower sensitivity when compared to nested PCR and qPCR. What is clear from this meta-analysis is that molecular-based technology has superior diagnostic accuracy over traditional methods of diagnosis. However, the small number of LAMP-based studies that were available for inclusion in this meta-analysis may potentially skew our view of the true diagnostic accuracy of LAMP over PCR-based assays.

According to Jamil *et al*. [49], clinical samples can contain lower levels of bacterial DNA, and this can affect the diagnostic sensitivity of molecular assays. Furthermore, biological samples may contain inhibitors could interfere with the diagnostic assay, negatively affecting accuracy. These inhibitors could interfere with cell lysis, thus making the extraction of DNA from cells difficult or degrade DNA and/or inhibit enzyme activity hindering amplification of DNA [50]. Consequently, when the performance of an index text is put under the microscope, it is imperative that the role of sample choice in the diagnostic process is equally considered.

In this study, pooled diagnostic sensitivity estimates were higher when serum and tissue samples were used to detect *B. abortus* while the same was significantly lower when blood, milk, and reproductive fluids were used. According to Parthiban *et al*. [38], whole blood can contain inhibitors such as hemoglobin, anticoagulants, and host cell DNA, which are likely to reduce the diagnostic sensitivity of an assay. These inhibitory effects were demonstrated by O’Leary *et al*. [3] when blood samples that had tested positive for *Brucella* spp. by serological methods all tested negative by PCR. Milk is also considered a challenging biological sample to use due to the inhibitory effects of its fat, protein, and, calcium constituents [51]. Having used milk as choice samples for the detection of *Brucella* spp., O’Leary *et al*. [3] believe that the number of detectable bacteria in milk samples can be reduced significantly if bacteria are shed before sampling, thus undermining diagnostic efforts. In their study, O’Leary *et al*. [3] found that the detection of *Brucella* spp. in milk samples by PCR was significantly lower than detection by classical microbiological methods, which also yielded a small number of colony-forming units, suggesting lower amounts of detectable bacteria in sample. This could explain the low diagnostic sensitivity observed in this study when milk-based assays were evaluated. Obtaining milk samples are relatively easy and are a minimally invasive technique; therefore, in theory, milk would be an ideal sample choice for the detection of *Brucella* spp. infections. This opinion, however, is not supported by our data or that of the previous study [3], particularly because it is rather challenging to predict fluctuations in the bacterial loads within an infected host to maximize the chances of obtaining detectable amounts of bacteria in collected milk samples by sampling at opportune times. While serum and tissue samples may also contain inhibitory substances, Wareth *et al*. [28] provide evidence for the reliable application of serum samples in the diagnosis of bovine brucellosis, while according to O’Leary *et al*. [3], samples taken from lymph tissue are probably the most promising to use for molecular detection of bovine brucellosis but due to limitations associated with obtaining lymph tissue from live animals, serum is considered to be the best alternative in as far as sample choice is concerned.

We further investigated the role of target genes in the accuracy of molecular diagnostic assays for detection of *B. abortus*. The majority of available reports on molecular detection of bovine brucellosis suggest that the IS711 repetitive genetic element is the more promising target for *Brucella* species differentiation as well as for the differentiation of field and vaccine strains [3, 20, 28, 29]. While this may be so, our findings indicate that targeting the IS711 genetic element may prove highly specific for detection of *B. abortus* but has significantly lower detection sensitivity when compared to other target genes. Instead, our findings suggest that targeting the BruAb2_0168 locus is the better option, as indicated by higher diagnostic accuracy estimates (sensitivity 92.3% [95% CI 87.0–96.0%] and specificity 99.3% [95% CI 98.0–100.0%]). The idea that the target gene plays an important role in the diagnostic accuracy of an assay is supported by the findings of Mugasa *et al*. [36], who demonstrated a significantly higher PCR accuracy with the use of satellite genes for detection of human African trypanosomiasis rather than with other genes.

The consumption of unpasteurized milk and milk products is the primary mode of animal-to-human transmission for brucellosis [1]. Even though our findings suggest that milk may not be an ideal sample choice for molecular detection of brucellosis, it is imperative that the brucellosis status of milk and milk products be accurately and timeously assessed before they reach consumers [22]. This can be achieved by testing cattle using accurate molecular techniques that have been designed based on highly specific gene targets and tested and validated on suitable clinical samples. Timely and precise diagnosis plays a significant role in the control and eradication of bovine brucellosis as well as curbing unnecessary production and economic losses arising from the culling of infected herds and restrictions imposed on the trade of such animals. As suggested by literature and the findings of this review, molecular methods are pioneering the way to improved diagnostics of infectious diseases and these findings are corroborated by the findings of two systematic reviews that focus on the diagnosis of human visceral leishmaniasis and human African trypanosomiasis [36, 39]. We strongly believe that the findings of this study will encourage the adoption of molecular diagnostic techniques to supplement traditional methods for the diagnosis of bovine brucellosis.

## Conclusion

The findings of this study indicate that choice of index test, clinical sample type, and gene target play a role in the overall performance of a diagnostic test and therefore, need to be collectively evaluated when a new diagnostic assay is developed. In addition, these findings indicate that DNA-based molecular methods are more effective in the diagnosis of *B. abortus* infections than traditional methods. LAMP, specifically the LAMP assay reported by Karthik *et al*. [7], had a higher diagnostic accuracy and is therefore recommended for the molecular detection of *B. abortus* infections where LAMP is the desired assay. Where PCR and qPCR are preferred alternatives and where limitations associated with LAMP cannot be overcome, the PCR assay reported by Alamian *et al*. [12] and the qPCR assays reported by Dehkordi *et al*. [25] and Barkallah *et al*. [26] can be reliably applied in the accurate diagnosis of bovine brucellosis.

While LAMP appears to be the diagnostically superior assay based on this meta-analysis, we cannot ignore its tendency for false positive amplification which may be attributed among other things to the type of clinical sample used, thus decreasing the diagnostic specificity of the assay. The true superiority of LAMP over PCR-based technology can only be ascertained more precisely with the availability of more studies. Therefore, we recommend that the meta-analysis be revisited in the future to gain better insight into whether the full potential of LAMP as a rapid and robust diagnostic tool can be harnessed and applied as an alternative tool for the accurate diagnosis of bovine brucellosis. In the interim, we recommend the use of PCR-based technologies like those mentioned above for the rapid and precise diagnosis of bovine brucellosis. However, we are also mindful that the methodological qualities of these studies (and many others included in this review and meta-analysis) may be compromised; therefore, studies of higher quality are necessary for future evaluation.

## Data Availability

The datasets used and/or analyzed during the current study are included in this published article. Any additional data will be made available on reasonable request from the corresponding author.

## Authors’ Contributions

ES and OT: Conceived the idea and reviewed the manuscript. LM: Designed the study, retrieved the study articles, extracted and analyzed the data, and wrote the manuscript. TEO: Extracted and analyzed the data and reviewed the manuscript. All authors have read and approved the final manuscript.
